# The role of interleukin-37 and interleukin-38 in the development and remission of autism spectrum disorder: a comprehensive review of neuroinflammatory mechanisms and potential therapeutic implications

**DOI:** 10.3389/fimmu.2025.1716197

**Published:** 2025-12-05

**Authors:** Zakeya Al Rasbi, Hiba Orsud, Sumaya Hasan Zoughbor, Malak Hajar, Abdulla Mahboob, Bassem Sadek

**Affiliations:** 1 Department of Microbiology and Immunology, College of Medicine and Health Sciences, United Arab Emirates University, Al Ain, Abu Dhabi, United Arab Emirates; 2 Department of Pharmacology & Therapeutics, College of Medicine and Health Sciences, United Arab Emirates University, Al Ain, United Arab Emirates; 3 Zayed Bin Sultan Centre for Health Sciences, United Arab Emirates University, Al Ain, United Arab Emirates; 4 Department of Chemistry, College of Sciences, United Arab Emirates University, Al-Ain, United Arab Emirates

**Keywords:** interleukin-37, interleukin-38, autism spectrum disorder, neuroinflammation, microglia, cytokines, therapeutic targets

## Abstract

Autism spectrum disorder (ASD) is a complicated neurodevelopmental syndrome characterized by abnormalities in social communication, lack of interests, and repetitive behaviors. Increasing evidence from recent studies indicates that neuroinflammation and immunological dysregulation play essential roles in the pathogenesis of ASD. This review consolidates current knowledge on two anti-inflammatory cytokines of the IL-1 family, interleukin-38 (IL-38) and interleukin-37 (IL-37), which have recently emerged as essential modulators of neuroimmune mechanisms in ASD, highlighting their emerging roles in ASD pathogenesis and therapeutic potential. Based on a combination of clinical and experimental findings, IL-38 has been reported to exert anti-inflammatory effects by suppressing microglial activation and reducing the release of pro-inflammatory cytokines. Consequently, modulation of IL-38/IL-36R signaling axis appears to represent a crucial mechanism regulating neuroinflammation in brain regions relevant to autism. On the other hand, studies indicate that IL-37 exhibits a consistent upregulation in the brain tissues associated with ASD, functioning via IL-37/IL-18Rα/IL-1R8 pathway, where it inhibits cytokine synthesis, alters microglial polarization, and affects communication along the gut–brain axis. While these findings establish IL-38 and IL-37 as possible biomarkers for ASD diagnosis and treatment targets, these investigations are still emerging. This review establishes the foundation for understanding the growing importance of cytokines and highlights the requirement for further research to clarify their roles and to formulate potential treatment approaches for ASD.

## 1 Introduction

**Figure 1:**
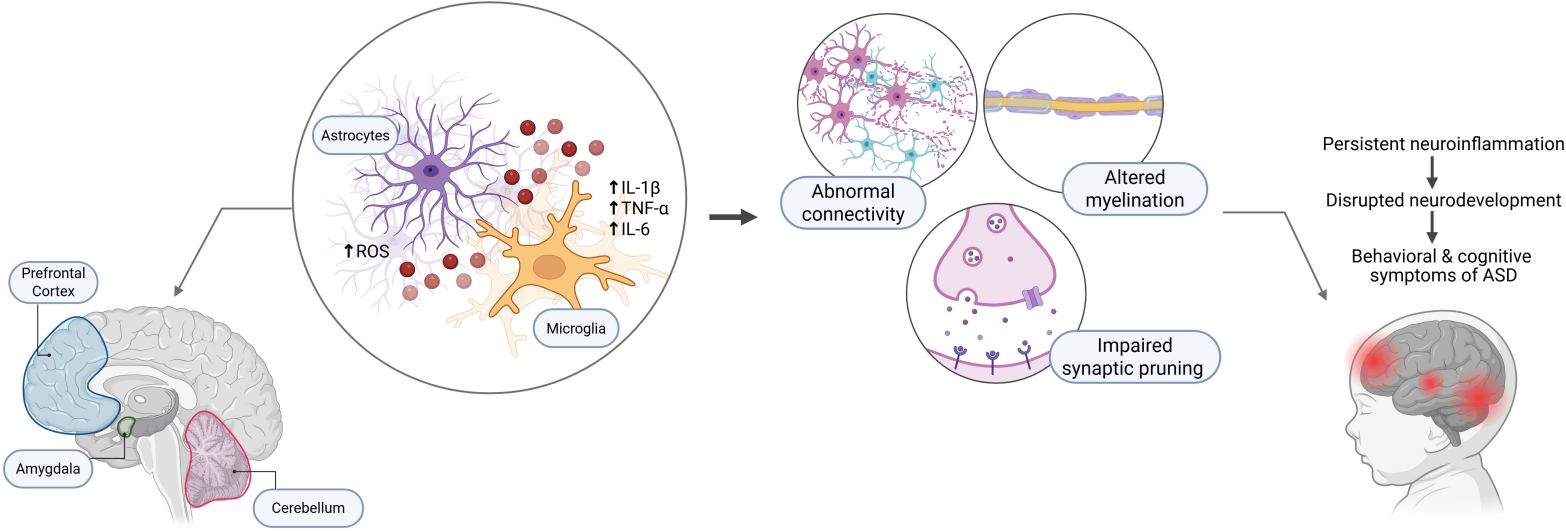
The neuroinflammatory hypothesis of autism. The figure illustrates the suggested cascade connecting neuroinflammation to the pathogenesis of ASD. In crucial brain regions associated with autism, such as the prefrontal cortex, amygdala, and cerebellum, persistent activation of astrocytes and microglia leads to increased production of reactive oxygen species (ROS) and pro-inflammatory cytokines, including IL-1β, TNF-α, and IL-6. These inflammatory mediators affect neuronal homeostasis and lead to several neuropathological consequences: abnormal neural connections, abnormal myelination, and defective synaptic pruning. These consequences collectively maintain persistent inflammation and impaired neurodevelopment, resulting in behavioral, sensory, and cognitive symptoms indicative of ASD.

Autism spectrum disorder (ASD) affects nearly one in 54 children in the United States, resulting in a considerable public health issue with significant economic and societal consequences (1). The disorder includes a diverse array of neurodevelopmental problems marked by enduring deficiencies in social communication and interaction, as well as confined and repetitive patterns of behavior, interests, or activities. Despite extensive research over several decades, the exact etiology of ASD remains unclear, with evidence indicating intricate interplay between genetic predisposition and environmental influences during crucial developmental phases (2). Recent decades have witnessed increasing evidence that has identified immunological dysfunction and neuroinflammation as significant factors in the pathophysiology of ASD. This paradigm shift from solely genetic or behavioral explanations to encompassing immune pathways has created new avenues for potentially addressing autism (3). Multiple studies have documented elevated levels of pro-inflammatory cytokines, including interleukin-1β (IL-1β), interleukin-6 (IL-6), interleukin-8 (IL-8), and tumor necrosis factor-α (TNF-α), in both peripheral blood and brain tissue of individuals with ASD (4, 5). These inflammatory markers correlate with symptoms’ severity, particularly in areas of communication impairment and aberrant behaviors, suggesting a direct link between immune system activation and core autism features (4–7). The neuroinflammatory hypothesis of autism as summarized in (**Figure 1**) has received considerable evidence from postmortem brain investigations that demonstrate active microglia and astrocytes in vital brain areas, such as the amygdala, cerebellum, and frontal cortex. Microglial activation, characterized by morphological alterations and increased production of inflammatory mediators, appears to be a consistent observation across many autism cohorts. This persistent neuroinflammatory condition may interfere with typical neurodevelopmental processes, including as synaptic pruning, neural connection, and myelination, hence contributing to the behavioral and cognitive symptoms of ASD (8). In this inflammatory context, anti-inflammatory cytokines have become of increased attention as possible protective agents or therapeutic targets. The interleukin-1 (IL-1) family, comprising both pro-inflammatory and anti-inflammatory constituents, has become notably pertinent to autism research (9). Among the anti-inflammatory cytokines in this family, interleukin-38 (IL-38) and interleukin-37 (IL-37) are among the most recently identified and least comprehended members; yet, preliminary data indicates that they may play a significant role in neuroinflammation associated with autism (10). IL-38, also known as IL-1F10, While, IL-37, known as IL-1F7, were first identified in 2001, 2000 respectively. However, they have only recently begun to be studied in the context of neurological disorders (11). IL-38 cytokine belongs to the IL-36 subfamily of the IL-1 family and exerts its anti-inflammatory effects primarily through competitive binding to the IL-36 receptor (IL-36R), thereby antagonizing pro-inflammatory IL-36 signaling (12). IL-37 mostly signals through the IL-18 receptor α (IL-18Rα) in association with IL-1 receptor 8 (IL-1R8, sometimes referred to as SIGIRR), resulting in considerable inhibition of inflammatory pathways (13). The discovery of IL-38 and IL-37’s potential relevance to autism came from ground-breaking research by Tsilioni and colleagues (2019, 2020), who demonstrated decreased IL-38 expression in the amygdala of children with ASD, coupled with its ability to inhibit microglial inflammatory responses. Besides, another study reported an increased IL-37 expression—particularly in the amygdala and dorsolateral prefrontal cortex—which represent critical areas for social behavior, emotional processing, and executive function, all domains significantly affected in autism. The significance of these findings extends beyond simple correlation, as the amygdala represents a critical brain region for social behavior, emotional processing, and fear responses—all areas significantly affected in autism. The amygdala’s involvement in autism is well understood, with neuroimaging studies repeatedly revealing structural and functional abnormalities in this region among individuals with ASD (9–14). The finding of the mentioned studies demonstrated that IL-38 is deficient in this autism-relevant brain region, whereas IL-37 is elevated, suggesting a potential mechanistic correlation between neuroinflammation and fundamental autism symptoms. The therapeutic possibilities of IL-38 and IL-37 research are particularly significant in consideration of the existing limitations in autism treatment. Although behavioral therapies constitute the standard for autism treatment, pharmaceutical alternatives are restricted and mostly target comorbid symptoms rather than fundamental characteristics. The recognition of the IL-37/38 axis as a potential biomarker and therapeutic target signifies a transformative shift toward precision medicine strategies in autism therapy (15). This review aims to comprehensively examine the current knowledge of the functions of IL-38 and IL-37 in autism spectrum disorder, integrating recent research findings to clarify their potential mechanisms of action, diagnostic significance, and treatment implications. And investigate the molecular biology of cytokines IL-37 and IL-38, their expression profiles in autism, their interactions with other immune mediators, and the implications for future research and clinical applications. This study aims to synthesize evidence from several research fields to position cytokines as significant factors in autism research and to underscore the potential of anti-inflammatory strategies in autism therapy.

This study provides the first comprehensive synthesis of IL-37 and IL-38 within a unified neuroimmune framework, contrasting with previous assessments that examined each cytokine separately. We propose a “IL-37/IL-38 axis” model of immune regulation in autism by analyzing their convergent and divergent signaling routes: IL-37 through IL-18Rα/IL-1R8 and IL-38 through IL-36R/IL-1RAPL1. This dual-cytokine viewpoint underscores the complementary anti-inflammatory and neuroprotective functions that have not been previously integrated within the context of ASD.

Additionally, IL-37 and IL-38 were selected together, as they form a complementary anti-inflammatory axis within the IL-1 family. IL-37 predominantly functions in intracellular inhibition through the IL-18Rα/IL-1R8 complex, while IL-38 modulates extracellular signaling via IL-36R and IL-1RAPL1. Examining both molecules concurrently facilitate a comprehensive knowledge of their synergistic regulation of neuroinflammation in autism.

## 2 Molecular biology and mechanisms

### 2.1 Structural characteristics and gene expression

Interleukin-38 (IL-38) and IL-37 are distinct members of the IL-1 cytokine family, characterized by their primarily anti-inflammatory functions and complex regulation mechanisms (16). The IL-38 gene is situated on human chromosome 2q13-14.1, positioned inside a cluster of other IL-1 family genes, indicating an evolutionary aggregation of anti-inflammatory mediators (17). This genomic structure illustrates the synchronized regulation of inflammatory responses and underscores the need of preserving immunological homeostasis via several redundant mechanisms (18). The IL-38 gene comprises five exons that extend over approximately 7.8 kilobases of genomic DNA, encoding a precursor protein of 152 amino acids with a molecular weight of 17 kDa (19). The protein structure is characterized by a high proportion of alanine, glutamic acid, and leucine residues, contributing to its unique three-dimensional conformation and receptor binding properties (20). While, IL-37 exists in five distinct isoforms (IL-37a through IL-37e), with IL-37b being the most extensively studied and biologically active variant. The IL-37b isoform consists of 218 amino acids and has a molecular weight of approximately 25 kDa in its precursor form (21). IL-37 requires proteolytic processing for activation of caspase-1 for full activation, linking its function directly to inflammasome activity as both a sensor and regulator of inflammatory responses (22), whereas IL-38 appears to be biologically active in its full-length form and secreted immediately upon synthesis, although truncated variants have been identified with potentially different functional properties (20). The tissue distribution of both cytokines is broad, with peak expression levels identified in the central nervous system. Their expression has been observed in various cell types, including neurons, astrocytes, and microglia, indicating multifaceted roles in neural function and immune regulation (13, 23). IL-38 is generated by fibroblast-like synoviocytes, keratinocytes, peripheral blood mononuclear cells, and CD19^+^ B cells, signifying its role in both innate and adaptive immune responses (24). The tissue distribution of IL-38 is extensive, with highest expression levels observed in the brain, heart, lungs, spleen, thymus, and skin (25). While, the tissue distribution of IL-37 is also broad, with expression detected in various immune and non-immune cells, including macrophages, dendritic cells, epithelial cells, and importantly for autism research, microglial cells in the central nervous system (26).

### 2.2 Intracellular mechanisms of action

**Figure 2:**
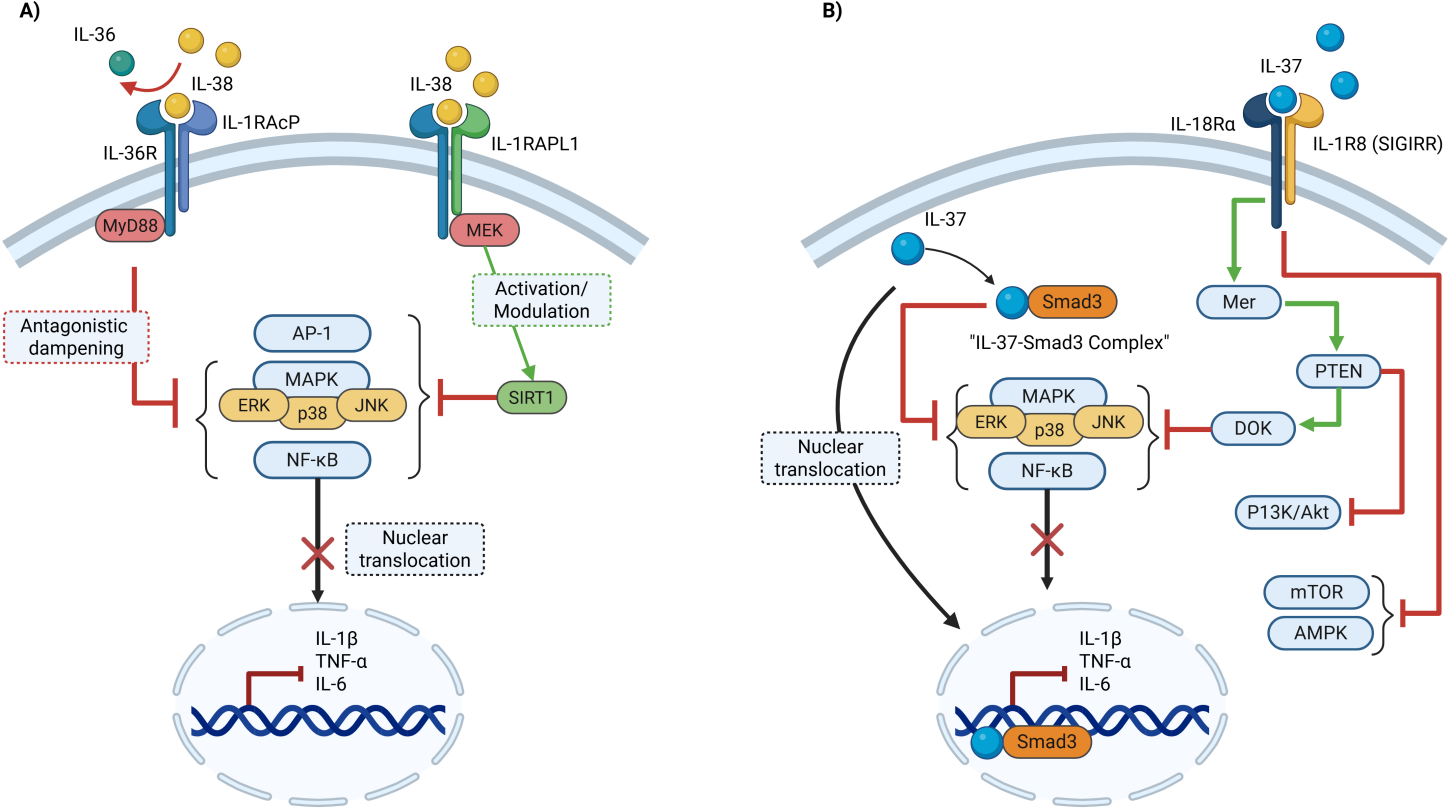
Schematic representation of the intracellular and extracellular signaling mechanisms of IL-38 and IL-37 and their relevance to autism spectrum disorder (ASD). **(A)** IL-38 binds to IL-36R/IL-1RAcP and IL-1RAPL1 receptors to modulate downstream inflammatory signaling. Through antagonistic dampening of IL-36R-mediated MyD88 recruitment and activation of SIRT1 via MEK, IL-38 inhibits the MAPK (ERK, p38, JNK) and NF-κB pathways, preventing NF-κB nuclear translocation and reducing transcription of IL-1β, TNF-α, and IL-6. **(B)** IL-37 exerts anti-inflammatory activity through two routes: formation of the IL-37–Smad3 complex, which translocates to the nucleus to suppress pro-inflammatory gene expression, and interaction with the IL-18Rα/IL-1R8 receptor complex, which activates the Mer–PTEN–DOK axis and inhibits PI3K/Akt, MAPK, and NF-κB signaling while modulating mTOR/AMPK activity.

The intracellular mechanisms of IL-38 are not as thoroughly defined as those of IL-37; nonetheless, growing data indicates that IL-38 demonstrates both pro-inflammatory and anti-inflammatory effects, depending on the environment. IL-38 can affect signaling pathways subsequent to its receptor contacts, influencing nuclear transcription factors and intracellular kinases. Upon binding of IL-38 to IL-36R in conjunction with IL-1 receptor accessory protein (IL-1RAcP), MyD88 is recruited, activating nuclear factor-κB (NF-κB) and mitogen-activated protein kinase (MAPK) pathways as illustrated in **Figure 2A**. Despite resembling a pro-inflammatory signaling pathway, the biological outcome is often governed by IL-38’s antagonistic function, which limits excessive inflammatory signaling and cytokine release (IL-1β, TNF-α, IL-6) (27). Furthermore, the interactions between IL-38 and IL-1RAPL1 has been associated with the modulation of intracellular stress and survival pathways. This involves the targeted recruitment of MEK and the activation of channel protein-dependent SIRT1, resulting in reduced activation of NF-κB, AP-1, and MAPK. IL-38 restricts the transcription of pro-inflammatory mediators, and indirectly promoting an M2 an anti-inflammatory phenotype linked to protection against neuronal damage in ASD-relevant brain regions (28).

Similarly, IL-37 exerts its intracellular anti-inflammatory activity through the Smad3-dependent pathway. IL-37 forms a cytoplasmic complex with Smad3 that translocates to the nucleus to suppress transcription of pro-inflammatory cytokine genes, as illustrated in **Figure 2B** (29). Additionally, intracellular IL-37 influences several critical signaling pathways involved in inflammation and cellular metabolism. The cytokine inhibits nuclear factor-κB (NF-κB) signaling, while also inducing metabolic reprogramming through mTOR inhibition and AMPK activation. in immune cells by inhibiting the mechanistic target of rapamycin (mTOR) pathway while simultaneously activating AMP-activated protein kinase (AMPK) (30). This metabolic shift creates a pseudo-starvational state that favors anti-inflammatory responses and limits the energy-intensive processes required for sustained inflammation. This mechanism is particularly relevant to autism research, as metabolic dysfunction has been implicated in ASD pathophysiology, suggesting that IL-37 helps restore immune-neural equilibrium (31).

### 2.3 Extracellular mechanisms of action

The biological activity of IL-38 is mostly mediated by interactions with extracellular receptors, corresponding to IL-37. Three principal receptor complexes have been identified: the IL-36 receptor (IL-36R), IL-1 receptor accessory protein-like 1 (IL-1RAPL1), and IL-1 receptor 1 (IL-1R1). The principal extracellular mechanism is competitive binding to IL-36R, wherein IL-38 functions as a natural antagonist that inhibits IL-36 cytokines from activating their pro-inflammatory pathways. This antagonism serves as a vital regulatory mechanism in tissues where IL-36 and IL-38 are co-expressed (32). IL-38 attenuates inflammation by interacting with IL-1RAPL1, hence modifying downstream pathways that diminish NF-κB, MAPK, and AP-1 activation. The receptor-mediated effects result in extensive suppression of cytokine and chemokine production, hence reducing the recruitment and activation of inflammatory cells. Moreover, IL-38’s extracellular signaling affects immune cell function. Through interaction with surface receptors, IL-38 reduces the release of pro-inflammatory mediators and promotes tolerogenic responses, hence enhancing its role as a regulator of chronic and sterile inflammation (33). Recent evidence further clarifies IL-38’s receptor versatility. in which, IL-36R, IL-38 binds with IL-1RAPL1 in neurons and astrocytes, influencing MAPK and NF-κB pathways in a cell-specific manner. IL-38 functions as a partial antagonist in the IL-1 family, attenuating the intensity of IL-36-mediated inflammation without completely inhibiting it. This fine-tuning impact differentiates IL-38 from IL-37, which widely inhibits cytokine transcription. In the central nervous system, IL-38 expression is confined to microglia and oligodendrocytes, suggesting context-specific neuroprotective functions (12).

Likewise, the extracellular signaling mechanisms of IL-37 have just recently been characterized and constitute similarly significant pathways for its anti-inflammatory actions (34). Extracellular IL-37 functions via a distinct receptor complex including IL-18 receptor α (IL-18Rα) and IL-1R8 (35). This receptor combination is distinctive among other IL-1 family members and provides specificity for the anti-inflammatory signaling of IL-37. The assembly of the IL-37/IL-18Rα/IL-1R8 complex triggers a cascade of anti-inflammatory signaling events (36). Upon binding to its receptor, IL-37 stimulates the Mer-PTEN-DOK pathway, resulting in the inhibition of various pro-inflammatory signaling cascades (37). This pathway entails the activation of Mer tyrosine kinase, which then activates PTEN (phosphatase and tensin homolog), resulting in the suppression of PI3K/Akt signaling and the activation of DOK (downstream of kinase) proteins (38). The extracellular signaling pathway also include the control of Toll-like receptor (TLR) responses, which are essential for the innate immune recognition of pathogen-associated molecular patterns (PAMPs) and damage-associated molecular patterns (DAMPs) (39). The binding of IL-37 to its receptor complex facilitates the recruitment of IL-1R8, which functions as a negative regulator of TLR signaling, therefore attenuating inflammatory responses to both infectious and sterile stimuli. Additionally, exogenous IL-37 modulates the polarization of macrophages and microglia toward an anti-inflammatory M2 phenotype, concurrently suppressing the pro-inflammatory M1 phenotype (40). The polarization shift is facilitated by the regulation of transcription factors including STAT6 and KLF4, which encourage M2 polarization, while concurrently suppressing NF-κB and IRF5, which boost M1 polarization (41). This process is especially relevant to autism studies, as microglial polarization has been associated with neurodevelopmental problems (35).

### 2.4 Preclinical and translational perspectives of IL-37 and IL-38 in ASD

Recent animal and preclinical research increasingly suggest the translational potential of IL-37 and IL-38 in modifying neuroinflammation In mouse models of systemic and neuroinflammation, the treatment of IL-37 markedly diminished pro-inflammatory cytokine levels (IL-1β, TNF-α, IL-6), reinstated microglial homeostasis, and enhanced behavioral and cognitive function (42). Similarly, IL-38 administration has demonstrated a reduction in glial activation, oxidative stress, and neuronal damage in animal models of encephalitis and ischemic brain injury (43). While direct ASD models that include these cytokines are scarce, similar observations in autoimmune and neurodegenerative diseases indicate their capacity to traverse the blood-brain barrier and diminish chronic neuroimmune activity (44). The data indicate that IL-37 and IL-38 could be effective therapeutic targets for re-establishing immunological equilibrium and mitigating inflammation-induced neuronal dysfunction in ASD. Additional translational research and preliminary clinical trials are necessary to assess their therapeutic potential and mechanistic specificity in human neurodevelopmental situations.

The molecular signaling processes described above constitute the mechanistic foundation for the modified expression patterns of IL-37 and IL-38 observed in individuals with ASD. Dysregulated cytokine signaling in brain and peripheral tissues likely contributes to the geographic variations observed in the amygdala and prefrontal cortex.

## 3 Expression patterns in ASD

### 3.1 Expression of brain tissue in ASD

The essential progression in understanding IL-38’s involvement in autism originated from the fundamental research conducted by Tsilioni and associates (2019; 2020), which provided the first direct evidence of altered IL-38 and IL-37 expression in brain tissue associated with autism (9, 14). This study utilized post-mortem brain samples from the University of Maryland NeuroBioBank to evaluate the gene expression of IL-38 and IL-37 in the amygdala of children with a confirmed diagnosis of autism spectrum disorder (ASD) against eight age-matched non-ASD controls. These findings showed intricate patterns of cytokine production that contradicted prior hypothesis regarding anti-inflammatory responses in autism. The results indicated a statistically significant reduction in IL-38 gene expression (p=0.001) in the amygdala of children with autism, signifying a substantial deficiency in this vital anti-inflammatory mediator inside a brain region integral to autism pathophysiology. The amygdala was chosen as the focal point of this study due to its recognized significance in autism. Neuroimaging research has consistently revealed structural and functional irregularities in the amygdala of individuals with ASD, encompassing modified volume, connection patterns, and activation responses to social stimuli (45). The amygdala is essential for processing emotional information, social signals, and fear responses—all areas profoundly impacted in autism (46). The identification of IL-38, a powerful anti-inflammatory cytokine, as missing in this area offers a reasonable molecular justification for the persistent neuroinflammation observed in autism brain tissue. Alongside the reduced IL-38 expression, the study indicated a significant drop in IL-36R expression (P = 0.04) among the identical amygdala samples (9). This finding is particularly important because IL-36R serves as the primary receptor for IL-38’s anti-inflammatory actions. The coordinated downregulation of both the cytokine and its receptor suggest a systemic impairment in the IL-38/IL-36R signaling axis within autism-affected brain tissue. This dual deficit may create a state of reduced anti-inflammatory capacity, leaving the tissue more vulnerable to inflammatory damage and less capable of resolving ongoing neuroinflammation. The implications of reduced IL-38 expression in the amygdala extend beyond simple inflammatory control. The amygdala plays critical roles in social behavior, emotional regulation, and sensory processing—all core areas of dysfunction in autism (47). Chronic neuroinflammation in this region, which is possibly intensified by IL-38 deficiency, may impair normal brain development and function, leading to the social communication difficulties and behavioral abnormalities associated with ASD (48). On the contrary, despite the chronic neuroinflammation associated with ASD, the study revealed a substantial increase in IL-37 expression (p=0.004) in the amygdala and dorsolateral prefrontal cortex of children with autism (14, 48). This finding was particularly striking because these brain regions are critical for social behavior, emotional processing, and executive function—domains that are characteristically impaired in ASD. The elevated expression of IL-37 seems to signify a compensatory anti-inflammatory reaction to the persistent neuroinflammation observed in these brain areas. Along with the heightened expression of IL-37, the study also demonstrated increased concentrations of the pro-inflammatory cytokines IL-18 and TNF-α in the corresponding brain areas. The co-expression of pro-inflammatory and anti-inflammatory mediators indicates a persistent inflammatory conflict inside the brain tissue of individuals with autism, with IL-37 possibly acting as an endogenous mechanism to regulate excessive inflammation. The coexistence of inflammatory and anti-inflammatory signals suggests that the immune response in ASD is marked not merely by excessive inflammation but by a complicated disruption of immunological homeostasis. The expression of IL-18 receptor α (IL-18Rα), a receptor for both IL-18 and IL-37, was similarly observed to be elevated in the same brain regions of children with ASD. This upregulation of the shared receptor suggests that both pro-inflammatory (IL-18) and anti-inflammatory (IL-37) signaling pathways are enhanced in autism brain tissue. The increased receptor expression may represent an attempt by the brain to respond more effectively to both inflammatory challenges and anti-inflammatory signals. Interestingly, the study also found that the expression of the NTR3/sortilin receptor was reduced in the amygdala and dorsolateral prefrontal cortex of children with ASD. This receptor is involved in neurotensin signaling, and its reduction may contribute to altered neuromodulation in autism. The relationship between reduced NTR3/sortilin expression and increased IL-37 expression suggests complex interactions between different signaling systems in the autism brain (14).

### 3.2 Compensatory mechanisms in the anti-inflammatory response of ASD

Tsilioni and colleagues (2019, 2020) demonstrated that both IL-37 and IL-38 are up-regulated in autistic brain regions and jointly suppress microglial inflammatory mediators via IL-18Rα/IL-1R8 and IL-36R pathways, they reported a reduced expression of IL-38 in brain tissue, accompanying a substantial elevation in IL-38 levels in the serum of children with ASD (P = 0.03) when compared to typically developing controls (9, 14). This contradictory discovery indicates the presence of compensation mechanisms via which the peripheral immune system attempts to prevent the inflammatory condition associated with autism by enhancing the production of anti-inflammatory mediators. The increased serum IL-38 levels in autism may indicate many underlying mechanisms. Initially, it may signify a systemic inflammatory response to persistent neuroinflammation, wherein peripheral immune cells elevate IL-38 production to offer anti-inflammatory assistance to the central nervous system. Secondly, elevated peripheral IL-38 may suggest compromised transport or use of this cytokine in the brain, requiring larger circulating levels to attain therapeutic concentrations in neural tissue (49). Additionally, they observed earlier that, increased expression of IL-37 in autism brain tissue can be understood as part of a broader compensatory anti-inflammatory response that attempts to counteract chronic neuroinflammation. This compensatory response appears to involve multiple anti-inflammatory mediators and regulatory mechanisms that are upregulated in response to persistent inflammatory stimuli. Nonetheless, despite these compensatory mechanisms, neuroinflammation exists in autism, indicating that the anti-inflammatory response is unable to completely regulate the inflammatory process. The insufficiency of the compensatory anti-inflammatory response in autism may originate from various sources, including genetic differences that influence anti-inflammatory mediator efficacy, excessive inflammatory stimuli, or deficiencies in inflammatory resolution pathways. Understanding the reasons behind the inadequacy of the compensatory response in managing inflammation in autism is essential for developing effective treatment strategies (50). These findings are summarized for clarity in **Table 1**.

**Table 1:** Expression patterns of IL-37 and IL-38 in autism spectrum disorder (ASD).

Cytokine/Receptor	Brain expression in ASD	Serum expression in ASD	Associated Receptors/Changes	Implications
IL-38	↓ Statistically significant reduction in amygdala (p=0.001), deficiency in vital anti-inflammatory mediator	↑ Substantial elevation in serum (p=0.03)	↓ IL-36R expression in amygdala (p=0.04)	Impaired IL-38/IL-36R axis, reduced anti-inflammatory capacity, persistent neuroinflammation; serum rise suggests compensatory mechanism
IL-37	↑ Substantial increase in amygdala and dorsolateral prefrontal cortex (p=0.004)	Not reported in this section	↑ IL-18Rα expression (shared receptor with IL-18)	Compensatory anti-inflammatory response; coexists with ↑ IL-18 and TNF-α, indicating inflammatory conflict
Other receptors	↓ NTR3/sortilin in amygdala & dorsolateral prefrontal cortex	—	Involved in neurotensin signaling	Reduction may contribute to altered neuromodulation; interacts with IL-37 upregulation

### 3.3 Developmental timing and critical periods

The correlation between fluctuations in parallel IL-38 and IL-37 expression and autism development constitutes a pivotal domain for future research. Current research indicates that immunological dysfunction in autism may initiate during prenatal development, with maternal immune activation being an important risk factor for ASD in offspring (51–53). The functions of IL-38 and IL-37 during these initial developmental stages are predominantly unexplored; however, they may be vital for comprehending the cause of autism. Research with animals has shown that inflammatory challenges during pregnancy can result in autism-like behaviors in the offspring, partly mediated by altered cytokine signaling and microglial activation (26, 54). The potential function of IL-38 and IL-37 in moderating early inflammatory responses may be substantial, especially considering their potent anti-inflammatory effects and capacity to modulate microglial activity (54). Insufficient expression or function of IL-38 and IL-37 during crucial developmental periods may render the developing brain vulnerable to inflammatory injury and consequent neurodevelopmental disorders. The neonatal period is a crucial phase for IL-38 function, characterized by significant synapse pruning and brain circuit remodeling (55). Microglia are crucial in these activities, and their functionality is markedly affected by the surrounding inflammatory microenvironment (56, 57). Dysregulated production of IL-38 and IL-37 during this phase may induce excessive microglial activation, leading to abnormal synaptic pruning and altered neuronal connection patterns associated with autism (58).

Therefore, the expression patterns of IL-38 and IL-37 in ASD expression imbalances contribute overall immunological dysregulation in ASD, wherein microglial activation and persistent inflammatory signals impair neural connections and behavioral results.

## 4 Immune dysregulation mechanisms in ASD

### 4.1 The neuroinflammatory landscape in ASD

Autism spectrum disorder is progressively acknowledged as an illness marked by persistent neuroinflammation, with numerous evidential sources endorsing this perspective. Postmortem brain investigations have repeatedly demonstrated active microglia and astrocytes in critical brain regions of individuals with ASD, including the amygdala, prefrontal cortex, and cerebellum (59). These inflammatory alterations are associated with increased concentrations of pro-inflammatory cytokines, chemokines, and other immune mediators that might interfere with normal neurodevelopmental processes. The inflammatory profile in autism is marked by an abundance of pro-inflammatory mediators, such as increased levels of IL-1β, IL-6, IL-8, TNF-α, and interferon-γ (60). Cytokines have been identified in both brain tissue and peripheral blood of individuals with ASD, indicating a systemic inflammatory condition that transcends the central nervous system (61). The severity of inflammatory markers often correlates with autism symptom severity, particularly in domains of social communication and repetitive behaviors, indicating a direct relationship between immune dysfunction and core autism features (62). In an inflammatory microenvironment, the function of anti-inflammatory mediators is essential for preserving immunological homeostasis and averting excessive tissue damage. The equilibrium between pro-inflammatory and anti-inflammatory signals dictates the overall inflammatory condition and its effect on brain function (63). The emergence of IL-38 and IL-37 as significant anti-inflammatory mediators in this environment places them as possible essential regulators of autism-related neuroinflammation.

### 4.2 Microglial activation and IL-38 regulation

Microglia, innate immune cells of the central nervous system, act as the principal mediators of neuroinflammation in autism. Under typical conditions, microglia maintain a surveillant state, persistently observing the cerebral environment for indications of injury or infection (64). I The activation of microglia in autism may be instigated by various reasons, including genetic predisposition, environmental chemicals, infections, and stress. In autism, microglia seem to be persistently activated, exhibiting modified morphology marked by larger cell bodies, thicker processes, and heightened expression of activation markers such as CD68 and Iba1 (35, 65). This activated state correlates with heightened synthesis of pro-inflammatory mediators, such as IL-1β, TNF-α, CXCL8, and nitric oxide, which can impair neurons and disturb normal cerebral function, establishing a cycle of inflammatory activation that may endure long after the initial stimulus has been eliminated. The involvement of IL-37 and IL-38 in modulating microglial activation is a crucial mechanism for regulating neuroinflammation in autism. Experimental studies have shown that IL-38 pre-treatment can markedly suppress neurotensin-induced microglial activation, decreasing the generation of pro-inflammatory mediators by as much as 90% at optimal concentrations (9). This inhibitory effect is mediated through IL-38’s binding to IL-36R on microglial cell surfaces, which prevents the activation of downstream inflammatory signaling pathways (12). The potency of IL-38’s anti-inflammatory properties on microglia is very significant. At doses as low as 1 ng/mL, IL-38 can effectively suppress the production of inflammatory mediators and exhibit greater effectiveness than IL-37; another anti-inflammatory cytokine within the IL-1 family. This high potency suggests that even modest increases in IL-38 availability could have significant therapeutic benefits for controlling autism-related neuroinflammation (9).

**Figure 3:**
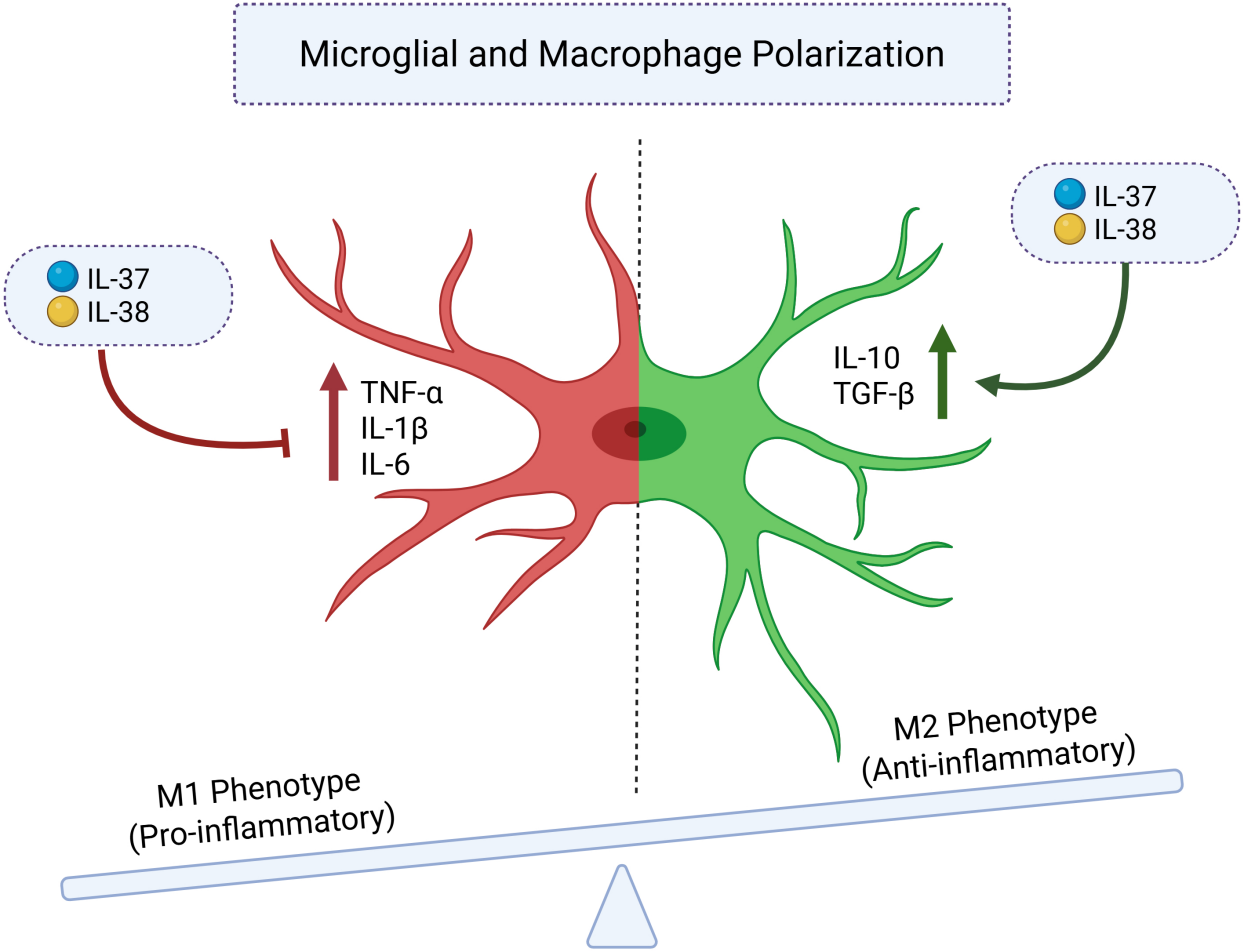
Microglial and macrophage polarization regulated by IL-37 and IL-38 in autism spectrum disorder (ASD). This schematic illustrates how IL-37 and IL-38 influence the polarization balance between the M1 pro-inflammatory phenotype and the M2 anti-inflammatory phenotype of microglia and macrophages. In ASD, chronic activation of M1 microglia leads to increased secretion of TNF-α, IL-1β, and IL-6, promoting oxidative stress and neuroinflammation that disrupt synaptic pruning and neural connectivity. IL-37 and IL-38 act as anti-inflammatory mediators of the IL-1 cytokine family that inhibit M1 activation while promoting M2 polarization. Through signaling via IL-18Rα/IL-1R8 (for IL-37) and IL-36R/IL-1RAPL1 (for IL-38), both cytokines suppress NF-κB and MAPK pathways and enhance the expression of anti-inflammatory mediators such as IL-10 and TGF-β. This shift toward the M2 phenotype facilitates the resolution of inflammation, supports tissue repair, and may contribute to restoring neuroimmune homeostasis in ASD.

Furthermore, one of the most important findings regarding IL-37’s role in autism relates to its ability to modulate microglial function and inflammatory responses. Due to their ability to suppress specific inflammatory mediators, such as IL-1β and CXCL8 (IL-8), which were particularly effective at addressing the type of neuroinflammation observed in ASD. The mechanism by which IL-37 inhibits microglial activation involves interference with key inflammatory signaling pathways. IL-37 apparently inhibits the NF-κB signaling pathway, which is crucial for the transcriptional activation of pro-inflammatory genes in microglia. This suppression leads to diminished production of inflammatory mediators that may affect neuronal function and contribute to the pathogenesis of neurodevelopmental disorders such as autism. Moreover, IL-37’s influence on microglia spans conventional anti-inflammatory effects, encompassing the facilitation of microglial polarization toward an M2 (alternatively activated) phenotype. M2 microglia facilitates tissue healing, neuroprotection, and the resolution of inflammation, whereas M1 microglia exacerbates inflammation and contributes to tissue damage. IL-37’s potential to induce M2 polarization indicates that it may not only inhibit detrimental inflammation but also facilitate brain repair and maintain homeostasis (**Figure 3**) (14).

### 4.3 The IL-38/IL-36R signaling axis in ASD

The IL-38/IL-36R signaling axis constitutes a vital regulatory route for modulating neuroinflammation in brain regions relevant to autism. Under typical circumstances, this system functions as a natural inhibitor of inflammatory reactions, averting excessive activation that may harm neural tissue. The simultaneous downregulation of IL-38 and IL-36R expression in autism brain tissue indicates a significant deficiency in this protective mechanism. The IL-36R receptor is present on various cell types in the central nervous system, such as microglia, astrocytes, and neurons (12). Upon binding to IL-36R, IL-38 acts as a competitive antagonist, inhibiting the attachment of pro-inflammatory IL-36 cytokines and obstructing their downstream signaling effects. This competitive inhibition is especially significant in relation to autism, because increased quantities of pro-inflammatory cytokines may surpass the system’s anti-inflammatory capabilities (32). The dysfunction of the IL-38/IL-36R axis in autism may play a role in various significant pathophysiological characteristics of the condition. Initially, decreased anti-inflammatory signaling may result in prolonged microglial activation, leading to the persistent synthesis of neurotoxic mediators (14, 58). Secondly, compromised IL-38 signaling may influence synaptic pruning mechanisms, as microglia are essential for the removal of synapses during development. Excessive or improper synaptic pruning, presumably caused by excessive microglial activation, has been suggested as a reason for the irregular connection patterns observed in autism (66).

### 4.4 Interaction with other inflammatory pathways

IL-38’s anti-inflammatory effects in autism encompass not just direct microglial control but also interactions with other significant inflammatory pathways associated with the illness. The complement system, demonstrated to be dysregulated in autism, is a way through which IL-38 may provide protective benefits (67). Activation of complement cascade may result in synaptic elimination and neuronal injury, processes potentially regulated by the anti-inflammatory effects of IL-38 (12). The interplay between IL-38 and other cytokines within the autism inflammatory network is sophisticated and multifarious. IL-38 can modulate the synthesis of additional anti-inflammatory mediators, including as IL-10 and transforming growth factor-β (TGF-β), thereby orchestrating an integrated anti-inflammatory response (68). In contrast, the persistent inflammatory condition in autism may inhibit IL-38 expression via negative feedback processes involving pro-inflammatory cytokines such as TNF-α and IL-1β (69). Research indicates that neurotensin, together with the pro-inflammatory cytokines IL-1β and TNF-α, can elevate IL-37 gene expression in cultured human microglia (70). This increase signifies an additional negative feedback mechanism in which inflammatory stimuli induce the synthesis of anti-inflammatory mediators to curtail excessive inflammation. In healthy individuals, this feedback mechanism ensures that inflammatory reactions are self-regulating and do not result in excessive tissue damage. In autism, this regulating system may be compromised by chronic inflammatory stimulation, resulting in persistent neuroinflammation despite elevated IL-37 expression (42). The transcriptional regulation of IL-37 involves multiple signaling pathways, including NF-κB, AP-1, and STAT pathways. The same pathways regulate pro-inflammatory cytokines, indicating a strong association between IL-37 expression and inflammatory responses. Comprehending the regulatory processes governing IL-37 expression may yield insights toward augmenting its therapeutic anti-inflammatory benefits (71).

### 4.5 Maternal immune activation and developmental programming

Maternal immunological activation, is a recognized risk factor for autism, presents an additional scenario in which IL-38’s protective effects may be significant (51, 52). Epidemiological studies have consistently demonstrated that maternal illnesses during pregnancy, especially in the second term, correlate with a heightened risk of ASD in infants (72). This association has been replicated in animal models, where maternal immune activation with viral mimetics or bacterial endotoxins leads to offspring with autism-like behaviors and neuroinflammation (73, 74). The mechanisms by which maternal immune activation elevates ASD risk entail the transference of inflammatory mediators from mother to fetus, potentially disrupting typical brain development. During pregnancy, maternal inflammatory responses can affect fetal brain development through the transfer of inflammatory mediators. particularly IL-6 and IL-17 can across the placenta barrier and directly affect fetal brain development (75). These cytokines can alter neuronal migration, synaptogenesis, and the development of neural circuits involved in social behavior and communication (76). IL-38’s ability to regulate maternal immune responses and potentially protect the developing fetal brain from inflammatory damage represents an important area for future investigation (77).

### 4.6 Peripheral immune dysfunction

Neuroinflammation is a key aspect of ASD pathogenesis, although peripheral immune dysfunction is also significant and may exacerbate central nervous system inflammation through various mechanisms. Individuals with ASD often have dysfunction in peripheral immune cell activity, characterized by modified T cell activation, biased cytokine production, and compromised regulatory processes (78). T-cell dysfunction in ASD is marked by heightened activation of pro-inflammatory Th1 and Th17 cell subsets, but regulatory T cells (Tregs) may be diminished in quantity or efficacy. This imbalance results in elevated production of pro-inflammatory cytokines, including interferon-γ, IL-17, and TNF-α, but anti-inflammatory cytokines such as IL-10 and TGF-β may be diminished. The resultant inflammatory condition can impact several organ systems and may exacerbate the gastrointestinal symptoms frequently seen in patients with ASD. Furthermore, B cell functionality is affected in ASD, exhibiting increased autoantibody synthesis and altered antibody responses (79). Certain individuals with ASD generate autoantibodies targeting brain proteins, such as neurotransmitter receptors and synaptic proteins, potentially leading to neuronal dysfunction. The existence of these autoantibodies indicates that molecular mimicry or other autoimmune pathways may play a role in the pathophysiology of ASD. Nonetheless, the functionality of Natural Killer (NK) cells is often affected in individuals with ASD, exhibiting reduced cytotoxic activity and altered cytokine synthesis. Natural Killer (NK) cells are crucial for immunological monitoring and the eradication of infected or abnormal cells; their failure may lead to heightened vulnerability to infections and possibly to the accumulation of damaged cells in the brain (80).

### 4.7 Blood-brain barrier in ASD

The blood-brain barrier (BBB) is essential for controlling the movement of immunological mediators between peripheral circulation and the central nervous system. Research indicates that in autism, the integrity of the blood-brain barrier may be impaired, potentially influencing the movement of pro-inflammatory and anti-inflammatory mediators (81). This malfunction might explain the puzzling observation of increased serum IL-38 levels coupled with decreased brain tissue expression in patients with ASD. Disruption of the blood-brain barrier in autism may stem from chronic inflammation, genetic predispositions, or environmental causes (82). When the barrier is breached, peripheral inflammatory mediators can more readily infiltrate the brain, potentially intensifying neuroinflammation (83). The transport of anti-inflammatory mediators such as IL-38 from the periphery to the brain may be disrupted, lowering their protective effects in critical areas. Comprehending the processes of IL-38 movement across the blood-brain barrier is essential for formulating successful therapeutic strategies (67). If IL-38/IL-37 transport fails in autism, therapeutic strategies might be needed to concentrate on improving barrier function, creating alternate delivery mechanisms, or directly targeting cerebral tissue with IL-38/IL-37 or their analogs (84). The temporal patterns of blood-brain barrier disruption in autism also merit attention. Early breakdown of the barrier may contribute to the initial inflammatory insult that initiates autism etiology. Conversely, if BBB failure arises from chronic neuroinflammation, it may serve as a secondary component that sustains the inflammatory condition by obstructing efficient resolution (85).

### 4.8 Gut-brain axis in ASD

Considering the significance of the gut-brain axis in the pathophysiology of autism, the potential involvement of IL-37 in controlling gastrointestinal inflammation and its impact on cerebral function constitutes a critical field of research, whereas the contribution of IL-38 in this axis is comparatively less explored. IL-37 has demonstrated protective effects in multiple forms of intestinal inflammation, indicating its potential for preventing gastrointestinal symptoms frequently observed in patients with ASD. The anti-inflammatory properties of IL-37 in the gastrointestinal tract may diminish intestinal permeability and restrict the translocation of bacterial products that can incite systemic inflammation. IL-37 may preserve intestinal barrier integrity, so mitigating systemic inflammatory responses that can exacerbate neuroinflammation in autism. This mechanism signifies a crucial connection between the peripheral and cerebral anti-inflammatory actions of IL-37 (86, 87). IL-37 may also affect the composition and function of the gut microbiome, which promotes the proliferation of beneficial bacteria while suppressing harmful species. The cytokine’s anti-inflammatory properties may foster a more favorable environment for beneficial bacteria, which might generate anti-inflammatory metabolites such short-chain fatty acids (88, 89). These metabolites can subsequently penetrate the circulatory system to the brain and contribute to the inhibition of neuroinflammation. The bidirectional characteristics of the gut-brain axis indicate that IL-37’s influence on gastrointestinal inflammation may directly impact brain function and behavior (89). IL-37 might improve gastrointestinal symptoms and behavioral outcomes in individuals with ASD by mitigating intestinal inflammation and maintaining a healthy gut flora. The dual effect renders IL-37 a compelling treatment target for tackling the intricate, multi-system characteristics of autism (90).

## 5 Potential therapeutic implications and clinical applications

### 5.1 IL-38 and IL-37 as potential therapeutic targets

The observation of IL-38 and IL-37 deficiency in brain tissue from individuals with autism, along with their significant anti-inflammatory effects, suggests that these cytokines may serve as viable therapeutic targets for autism spectrum disorder (12, 91). The therapeutic potential of this pair of cytokines is supported by several key factors: their natural existence in the human body, their established safety profile in preliminary studies, the potency of IL-38 at low concentrations and its receptor specificity, along with the extensive anti-inflammatory profile of IL-37, has shown safety in initial studies (12, 15, 32, 40, 71, 91). Current treatments for autism predominantly emphasize behavioral interventions and symptomatic management, while pharmacological options addressing the core features of the disorder remain limited. Most medications utilized in the treatment of autism target comorbid conditions, including anxiety, attention deficits, and aggressive behaviors, rather than addressing the underlying pathophysiology (92). Therapies based on IL-38 and IL-37 may signify a shift toward precision medicine strategies aimed at addressing the specific biological mechanisms associated with autism (42, 67–93). The development of cytokine-based therapeutic agents may manifest in various forms. The direct administration of recombinant IL-38 or IL-37 is a straightforward approach, potentially delivered via intravenous, subcutaneous, or intranasal routes (36, 94, 95). The intranasal route is advantageous for neurological applications due to its ability to avoid the blood-brain barrier, facilitating direct delivery of therapeutic agents to the central nervous system (94). Alternative approaches may include the creation of cytokine intervention analogs or mimetics that exhibit improved stability, potency, or brain penetration characteristics (96, 97). Small molecule agonists or mimetics that target the IL-38/IL-36R pathway or the IL-18Rα/IL-1R8 axis for IL-37 may offer oral bioavailability and enhanced pharmacokinetic properties relative to protein-based therapeutics (97). Gene therapy methods utilizing viral vectors for the direct delivery of cytokine expression constructs to targeted brain regions present a viable strategy; however, this involves comprehensive safety evaluations (98).

### 5.2 Mechanisms of potential therapeutic action

Therapies based on IL-37 and IL-38 are expected to provide therapeutic benefits through various complementary mechanisms that target different facets of ASD pathogenesis (99). The principal method is the direct attenuation of neuroinflammation via their common capacity to limit the production of pro-inflammatory cytokines (e.g., IL-1β, TNF-α, IL-6, and chemokines) and to enhance anti-inflammatory responses. Both cytokines can reduce the chronic inflammatory environment linked to autism by promoting a more conducive environment for typical neurodevelopment and synaptic function (67).

The regulation of microglial and macrophage activation constitutes a significant therapeutic strategy. Both IL-37 and IL-38 facilitate polarization toward the M2 (anti-inflammatory) phenotype while suppressing excessive M1 activation, thus reinstating homeostatic immune activities. This effect is especially pertinent for enhancing synaptic pruning and brain connection, functions that are impaired in ASD (100, 101).

The impact on the gut–brain axis amplifies therapeutic potential. IL-37 has demonstrated efficacy in diminishing intestinal inflammation and fostering a healthy gut microbiota (70), whereas IL-38 modulates epithelial and mucosal inflammation via antagonism of IL-36R and interaction with IL-1RAPL1 (102). By disrupting the cycle of peripheral inflammation that induces neuroinflammation, both cytokines may enhance behavioral symptoms and gastrointestinal comorbidities in patients with autism (103).

A fundamental difference is in the metabolic impacts. IL-37 is known to facilitate the activation of AMP-activated protein kinase (AMPK) and to inhibit mechanistic target of rapamycin (mTOR) signaling, so inducing a pseudo-starvation state that restricts energy-demanding inflammation and enhances mitochondrial function (13). IL-38, albeit less researched in this context, indirectly affects cellular metabolism by inhibiting the NF-κB, MAPK, and AP-1 pathways and enhancing SIRT1 expression. These effects may focus on enhancing cellular stress responses, while IL-38’s function in direct metabolic reprogramming has yet to be completely elucidated (104).

IL-37 and IL-38 collectively offer comparable therapeutic strategies, focusing on cytokine suppression, immune cell polarization, and control of the gut–brain axis, while differing in receptor engagement and degree of metabolic reprogramming (32, 95). The convergence and differentiation of these activities underscore the possibility of both cytokines as viable immunoregulatory approaches in ASD and associated neuroinflammatory disorders (67).

### 5.3 Biomarker development and diagnostic applications

Besides contributing to its therapeutic potential, both IL-37 and IL-38 exhibit promise as biomarkers for the diagnosis and monitoring of autism treatment (94). Contemporary diagnostic procedures predominantly depend on behavioral evaluations, which are inherently subjective and frequently postponed until pronounced symptoms manifest (95). Biological markers facilitating earlier and more objective diagnoses would signify a significant advancement in autism care (15, 32). The expression patterns of IL-38 in autism—typically diminished in brain tissue but elevated in serum—indicate its potential as an accessible biomarker. Increased blood levels may be utilized for diagnosis or therapy response monitoring; however, specificity must be confirmed against other inflammatory disorders by extensive comparative investigations (12). Likewise, IL-37 concentrations in peripheral blood, in conjunction with associated cytokines and systemic inflammatory indicators such C-reactive protein, may assist in stratifying patients based on inflammatory status and potential treatment response (105). Both cytokines may function as dynamic indicators of therapy success. Alterations in circulating IL-37 or IL-38 post-intervention may offer objective metrics that enhance behavioral evaluations, especially for assessing anti-inflammatory or immunomodulatory therapies (106). Analysis of cerebrospinal fluid may provide a more direct evaluation of central nervous system levels; nevertheless, its invasive nature restricts normal clinical use. Advanced neuroimaging techniques, such as PET tracers for active microglia, may non-invasively evaluate neuroinflammation and indirectly indicate IL-37/IL-38 activity (107). Genetic biomarkers may enhance patient selection, particularly polymorphisms in genes associated with IL-37 or IL-38 signaling, inflammatory control, or autism susceptibility (108). Pharmacogenomic profiling may enhance therapeutic dosage and reduce side effects. Composite biomarker panels that integrate inflammatory, neuroimaging, genetic, and clinical data may offer the most precise framework for identifying patients who are most likely to benefit from cytokine-based therapies and for monitoring their treatment progress.

### 5.4 Combination therapies and synergistic approaches

Due to the intricate, multifactorial characteristics of autism, interventions utilizing IL-37 and IL-38 will probably be most efficacious when incorporated into combination or multimodal strategies (109). Both cytokines may be used with additional anti-inflammatory medications, behavioral therapies, or metabolic modulators to attain synergistic advantages (110). The integration of other anti-inflammatory cytokines, such as IL-10, or pharmaceutical inhibitors of pro-inflammatory pathways (e.g., TNF-α blockers, IL-1β antagonists), may yield total control of inflammation. Nutritional therapies, such as omega-3 fatty acids, antioxidants, and anti-inflammatory diets, along with lifestyle changes like exercise and stress reduction, may augment the cytokine-mediated therapeutic response (111–113). Targeted neurological medicines, including glutamate receptor modulators, GABA enhancers, or mitochondrial support supplements, may be used with IL-37/IL-38 therapy to tackle both the inflammatory and non-inflammatory components of ASD pathogenesis (114). Sequential techniques may prove particularly efficacious; for instance, commencing treatment with IL-37 or IL-38 to mitigate neuroinflammation, subsequently followed by measures aimed at restoring synaptic function or neurotransmitter equilibrium (39, 42). Personalized medicine strategies will be crucial for enhancing these combination regimens (95). Therapy selection guided by biomarkers—utilizing serum cytokine levels, genetic profiles, or neuroimaging results—may identify patients most predisposed to respond to IL-37/IL-38, inform dose, and determine treatment duration (32). Consequently, IL-37 and IL-38 may function as both therapeutic agents and integral elements of personalized, multimodal therapy strategies for autism (110).

### 5.5 Possible personalized medicine approaches

The variability inherent in autism spectrum disorder indicates that personalized medicine strategies are crucial for enhancing treatment efficacy. Not all individuals with autism exhibit deficiencies in IL-38 or IL-37, and the severity of such deficiencies can considerably vary among patients (67). Identifying patients who are most likely to benefit from IL-38 or IL-37 therapy is essential for effective clinical implementation. Genetic testing may identify individuals with polymorphisms in IL-38, IL-37, or their receptors’ genes, such as IL-36R, IL-18Rα, and IL-1R8, which could influence cytokine function or expression (115). Utilizing comprehensive cytokine panels for inflammatory profiling may facilitate the characterization of each patient’s inflammatory signatures and inform personalized treatment strategies. Advanced neuroimaging techniques may facilitate direct evaluation of neuroinflammation in particular brain regions, thereby enabling targeted therapeutic strategies (116, 117). The timing of cytokine interventions may necessitate personalization according to individual developmental trajectories and symptom profiles. Early intervention during crucial developmental phases may yield significant advantages; however, determining the most effective treatment timing requires comprehensive research. Certain patients may derive advantages from continuous IL-38 supplementation, whereas others might need periodic treatment during phases of heightened inflammatory activity (97). Age-related factors will be significant for the personalization of IL-38 and IL-37 therapy. The developing brain may exhibit distinct responses to anti-inflammatory treatments relative to the mature brain, necessitating adjustments in dosing strategies (118). Long-term safety considerations are crucial for pediatric applications due to the chronic nature of autism and the potential requirement for prolonged treatment durations (119).

### 5.6 Challenges and limitations

Although IL-38 and IL-37 show therapeutic potential, numerous challenges need to be resolved prior to their successful application in clinical settings for autism treatment. The main obstacle pertains to drug delivery to the central nervous system, given that the blood-brain barrier restricts the penetration of numerous protein-based therapeutics. Establishing efficient delivery mechanisms to attain therapeutic levels of IL-38 or IL-37 in pertinent brain areas is crucial for clinical efficacy (120). Safety considerations represent an important challenge, especially in pediatric applications. IL-38 and IL-37 are naturally occurring cytokines with established anti-inflammatory properties; however, the long-term effects of their exogenous administration remain poorly understood. Concerns may encompass immune suppression, heightened vulnerability to infections, or disruption of normal immune development in children (41, 94, 121). The cost and complexity of protein-based therapeutics may restrict the accessibility and universal implementation of cytokine therapy. Establishing cost-efficient manufacturing methods and alternative formulations that lower treatment expenses is crucial for facilitating widespread patient access (122). Insurance coverage and reimbursement policies for innovative autism treatments might create obstacles to clinical implementation (123). Regulatory pathways for autism treatments face distinct challenges, as conventional clinical trial endpoints may insufficiently reflect the advantages of anti-inflammatory therapies (47). Establishing suitable outcome measures capable of identifying clinically significant enhancements in autism symptoms after IL-38 or IL-37 treatment is essential for regulatory approval. The variability of autism can complicate the design of clinical trials and the interpretation of their results (99). **Table 2** summarizes the therapeutic strategies of IL-37 and IL-38, highlighting their modes of administration, delivery approaches, mechanistic targets, metabolic effects, biomarker potential, and overall promise in ASD.

**Table 2:** Therapeutic strategies and clinical applications of IL-37 and IL-38 in ASD.

Feature	IL-37	IL-38
Direct administration	Recombinant IL-37; intravenous, subcutaneous, or intranasal routes	Recombinant IL-38; intravenous, subcutaneous, or intranasal routes
Alternative approaches	Cytokine analogs or mimetics targeting IL-18Rα/IL-1R8 axis; gene therapy constructs	Cytokine analogs or mimetics targeting IL-36R or IL-1RAPL1; gene therapy constructs
Delivery advantages	Intranasal delivery avoids the blood–brain barrier, facilitating direct access to CNS	Intranasal delivery also applicable; potency at low concentrations allows significant therapeutic effect
Mechanistic targets	Broad inhibition of pro-inflammatory cytokines (IL-1β, TNF-α, IL-6); promotes M2 polarization; regulates gut–brain axis	Potent suppression of microglial activation via IL-36R; antagonizes pro-inflammatory IL-36 signaling; interacts with IL-1RAPL1
Metabolic impacts	Induces metabolic reprogramming: activates AMPK, inhibits mTOR, creates pseudo-starvation state, enhances mitochondrial function	Indirectly affects cellular metabolism via inhibition of NF-κB, MAPK, AP-1, and by enhancing SIRT1 expression
Biomarker potential	Peripheral IL-37 concentrations and associated cytokines (e.g., C-reactive protein) may stratify patients and monitor therapy	Diminished brain expression but elevated serum levels may serve as accessible biomarker for diagnosis and treatment response
Therapeutic promise	Broad immunoregulatory, metabolic, and gut–brain effects suggest multi-system benefits in ASD	High potency at low doses, receptor specificity, and natural presence highlight potential for targeted therapy

In regard to the considerations for clinical implementation, while IL-37 and IL-38 exhibit potential in reducing neuroinflammation, translation must include blood-brain barrier delivery, the danger of immunosuppression, and long-term safety in juvenile populations. Feasible pathways encompass intranasal delivery, receptor-mediated transcytosis-engineered biologics, and nanocarriers/exosomes, each accompanied by pharmacokinetic/pharmacodynamic and target-engagement evaluations. Trials must focus on inflammatory endotypes, utilize validated clinical endpoints (ABC-I, SRS-2, RBS-R) in conjunction with objective measures (EEG, eye-tracking, specific neuroimaging), and establish comprehensive safety monitoring (infection indicators, ADA, growth/neurodevelopment). A tiered strategy—Phase I safety/pharmacokinetics, succeeded by biomarker-guided Phase Ib/IIa is viable and consistent with precision medicine methodologies in autism spectrum disorder.

## 6 Future research directions

### 6.1 Key research areas

The nascent field of IL-38 research in autism offers significant prospects for enhancing our comprehension of the illness and formulating innovative therapeutic strategies (41). Several priority research areas have been recognized that could profoundly influence the translation of IL-38 discoveries into therapeutic applications. Extensive replication studies are critically required to validate the preliminary observations of decreased IL-38 expression in the brain tissue of individuals with autism, alongside the elevated levels of IL-37 in the brains of children diagnosed with ASD. These investigations must encompass broad populations, various brain areas, and thorough characterization of inflammatory profiles to determine the generalizability and specificity of both cytokines in autism (67). Longitudinal studies monitoring variations in IL-38 and IL-37 expression over time may elucidate the temporal dynamics of neuroinflammation in autism and pinpoint essential intervention periods (47). Mechanistic investigations into the molecular processes associated with IL-38 deficit and the dysregulation of IL-37 in autism constitute another essential research goal (90). Determining whether IL-38 shortage arises from diminished production, enhanced degradation, compromised transport, or alternative mechanisms could guide therapeutic approaches (27). Additionally, comprehending the correlation between IL-37 expression and the progression of symptom severity over time may facilitate the identification of biomarkers for treatment efficacy. Furthermore, comprehending the interactions of IL-37 with various inflammatory mediators, neurotransmitter systems, and developmental processes in relation to autism will be essential for enhancing therapy strategies (32). Research into the regulation of cytokine gene expression in autism, encompassing epigenetic changes and transcriptional regulatory mechanisms, may uncover novel intervention targets. Animal model research will be crucial for evaluating IL-38 and IL-37-based treatment strategies and comprehending their mechanisms of action (24). Current autistic animal models, encompassing those derived from genetic abnormalities or maternal immune activation, may be utilized to assess the impact of cytokine supplementation on autism-like behaviors and neuroinflammation. These studies could also examine appropriate dose regimes, administration modalities, and timing of treatment to maximize therapeutic efficacy (111).

### 6.2 Clinical translation strategies for clinical development

The clinical development of IL-38 and IL-37-based therapies for autism requires a systematic approach to address the challenges and limitations discussed above. The translation of IL-38 and IL-37 research into clinical applications involves thoroughly planned studies that evaluate both safety and efficacy aspects. First Phase I clinical trials assessing the safety, tolerability, and pharmacokinetics of IL-38 or IL-37 in healthy volunteers and individuals with autism are essential preliminary steps. These studies must assess various delivery routes and dosing regimens to determine optimal treatment protocols. Concurrently, evolving biomarker development studies should be conducted alongside therapeutic development to establish methods for patient selection and treatment monitoring (67). Comprehensive inflammatory profiling of large autism cohorts may reveal subgroups that are most likely to benefit from IL-38/IL-37 therapy and facilitate the development of predictive algorithms for treatment response. Neuroimaging studies employing advanced techniques to evaluate neuroinflammation may yield objective assessments of treatment impacts on brain inflammation (124). The development of outcome measures designed to evaluate the impact of anti-inflammatory treatments in autism is a significant need. Traditional autism assessment tools may not be sensitive to the types of improvements expected from both cytokines therapy, necessitating the development of new instruments that can capture changes in inflammation-related symptoms. Cognitive and behavioral measures that are sensitive to neuroinflammatory changes could provide more appropriate endpoints for clinical trials (125). **Table 3** delineates the primary animal models of ASD that demonstrate neuroimmune alterations relevant to the investigation of IL-37 and IL-38. These models, including maternal immune activation (MIA), valproic acid (VPA) exposure, and BTBR mice, imitate critical characteristics like increased pro-inflammatory cytokines, microglial activation, and behavioral impairments. While direct research including IL-37 or IL-38 have not been conducted in these systems, they offer strong platforms for evaluating the anti-inflammatory, microglia-modulating, and gut–brain axis effects of these cytokines. The table emphasizes critical experimental methodologies—such as intranasal versus systemic delivery, microglial M1/M2 profiling, and behavioral rescue assays—that might connect molecular discoveries with translational results in ASD research.

**Table 3:** Summary of ASD-relevant animal models exhibiting neuroimmune alterations related to the investigation of IL-37 and IL-38.

Model	Core phenotype(s)	Neuroimmune features	Status for IL-37/IL-38	High-priority experiments
MIA (poly(I:C)/LPS)	Social/USV deficits, repetitive behavior	↑ IL-1β/IL-6/TNF-α; M1-microglia	No/limited direct tests	Preventive vs rescue dosing; intranasal vs systemic; microglial M1/M2, cytokines, three-chamber social test (126)
VPA prenatal	Social/repetitive, anxiety, gut changes	Microgliosis; barrier disruption	No/limited direct tests	IL-37: gut permeability (FITC-dextran), cytokines; IL-38: IL-36R signaling in microglia; behavior panel (127)
BTBR	Strong baseline inflammation	Astro/microgliosis; high cytokines	No direct tests	Chronic rescue; TSPO-PET (if available); Iba1/CD68, Arg1/iNOS, EEG/behavior (128)
Shank3	Synaptic deficits	Context-dependent microglia changes	No direct tests	Electrophysiology (LTP), dendritic spines, microglial phagocytosis indices ± IL-37/IL-38 (129)
CNTNAP2	Social/USV, seizures	Immune pathway shifts	No direct tests	Seizure safety, sleep/EEG, cytokines; dose-finding (130)
Fmr1/MeCP2	Cognitive/sensorimotor	Mild–moderate inflammation; metabolic	No direct tests	AMPK/mTOR readouts (IL-37), behavior and synaptic physiology (131)
Microbiome/dysbiosis	Variable	Barrier dysfunction; systemic→CNS	No direct tests	IL-37 barrier stabilization; cytokine panels; metabolomics (132)

### 6.3 Technological innovations and methodological advances

Research on IL-38 and IL-37 in autism will be enhanced by various technological innovations and methodological advancements. Advanced neuroimaging techniques capable of non-invasively assessing neuroinflammation and cytokine activity in living patients may significantly enhance our capacity to study and monitor IL-38 function in autism (12). Positron emission tomography (PET) tracers specific for IL-38 or IL-37 or their receptors could enable direct visualization the IL-38 and IL-37 system in the autism brain, and measurement of neuroinflammation and help to identify patients with the highest levels of brain inflammation (47). Single-cell sequencing technologies could provide unprecedented insights into cell-type-specific IL-38 or IL-37 expression patterns in autism brain tissue. These approaches could identify which specific cell populations are responsible for IL-38 deficiency as well as IL-37 dysregulation and reveal potential cellular targets for therapeutic intervention. Spatial transcriptomics techniques could map the precise anatomical distribution of these cytokines’ expression changes within autism-affected brain regions (16). Innovations in drug delivery are essential for addressing the limitations of the blood-brain barrier that prevent the delivery of protein therapeutics to the central nervous system (80). Nanotechnology strategies, including the use of nanoparticles and liposomes aimed at improving brain penetration through methods like intranasal delivery, may enhance the delivery of IL-38/IL-37 to target tissues. Focused ultrasound techniques capable of temporarily opening the blood-brain barrier in designated brain regions may facilitate the targeted delivery of IL-38 to areas related to autism (9, 133).

### 6.4 Further implications for ASD research

The emergence of IL-38/IL-37 as potential key players in autism pathophysiology has broader implications for autism research. This underscores the significance of neuroinflammation as a therapeutic target and emphasizes the potential of anti-inflammatory strategies for the treatment of neurodevelopmental disorders. Many neurodevelopmental conditions, such as attention deficit hyperactivity disorder, intellectual disability, and cerebral palsy, are associated with inflammatory processes that may be targeted through IL-37-based interventions (67). The success of IL-38 research may facilitate the exploration of additional anti-inflammatory cytokines and immune modulators in autism. The findings related to IL-38 demonstrate the importance of investigating rare or newly identified molecules in autism research. Several cytokines and immune mediators are inadequately characterized regarding neurodevelopmental disorders, and a systematic investigation of these molecules may uncover further therapeutic targets (9, 12). The integration of immunology and neuroscience approaches is crucial for enhancing our comprehension of autism pathophysiology. The implications of personalized medicine derived from cytokine research correspond with the broader trends toward precision approaches in autism treatment. As our comprehension of autism heterogeneity advances, the capacity to align specific treatments with individual patient profiles will gain significance. Research on IL-38 and IL-37 offers a framework for utilizing biological markers in treatment selection and monitoring (15, 47). The integration of inflammatory and metabolic pathways emphasized by IL-37 research may enhance our understanding of other conditions involving these interacting systems. The acknowledgment that anti-inflammatory interventions may significantly influence cellular metabolism and brain function has the potential to inform novel therapeutic strategies for a range of neurological and psychiatric disorders (134).

### 6.5 Summary and discussion

The characterization of IL-38 and of IL-37 in autism spectrum disorder indicate an important progression in our comprehension of the neuroinflammatory processes that underlie this complex neurodevelopmental illness. Decreased IL-38 expression in autism brain tissue, along with its significant anti-inflammatory effects and capacity to modulate microglial function, establishes this cytokine as a potential biomarker and treatment target (27, 41). The discovery that the brain proactively seeks to mitigate neuroinflammation by upregulating anti-inflammatory mediators such as IL-37 offers a novel paradigm for comprehending autism and formulating treatment strategies. The existing research, however restricted, offers persuasive evidence for the role of cytokines in the pathophysiology of autism. The coordinated downregulation of IL-38 and its receptor IL-36R in the amygdala of children with autism suggests a fundamental impairment in anti-inflammatory signaling within this critical brain region (12, 90). The paradoxical elevation of serum IL-38 levels suggests the presence of compensating mechanisms; however, these may be inadequate to address the shortage in the central nervous system. IL-38’s therapeutic potential is evidenced by its capacity to decrease microglial activation and limit the generation of pro-inflammatory markers associated with autism. The significant potency of IL-38, together with the broad anti-inflammatory properties of IL-37 and its natural presence in the human body, indicates that therapies utilizing these cytokines may offer effective and safe therapeutic alternatives for individuals with autism (47, 135, 136). Nonetheless, considerable obstacles persist prior to their successful implementation in clinical practice. This encompasses creating efficient delivery mechanisms to overcome blood-brain barrier limitations, determining safety profiles for prolonged usage, and formulating suitable clinical trials that can validate efficacy in the diverse autistic population. The expense and complexity of protein-based therapies provide significant practical challenges that require resolution (9, 24). In addition to, the existing evidence is limited by small cohort sizes with a concentration of data from a few research groups, particularly the investigations by Tsilioni et al. on post-mortem brain tissue. These data indicate a neuroprotective IL-37/IL-38 signature; however, validation in larger, multicenter cohorts incorporating cytokine profiling, neuroimaging, and behavioral endpoints is necessary. Future research should evaluate long-term cytokine dynamics and gene expression patterns to establish causality rather than just correlation. However, significant challenges remain before IL-38 can be successfully translated into clinical practice (12). These include developing effective delivery methods to overcome blood-brain barrier limitations, establishing safety profiles for long-term use, and designing appropriate clinical trials that can demonstrate efficacy in the heterogeneous autism population (137, 138). The cost and complexity of protein-based therapeutics also present practical barriers that must be addressed (139). Besides, significant challenges remain in translating IL-37 research into clinical applications; the rapid pace of discovery in this field provides reason for optimism (13). The convergence of advances in drug delivery technology, biomarker development, and personalized medicine approaches creates unprecedented opportunities for developing effective IL-37-based therapies (140).

**Figure 4:**
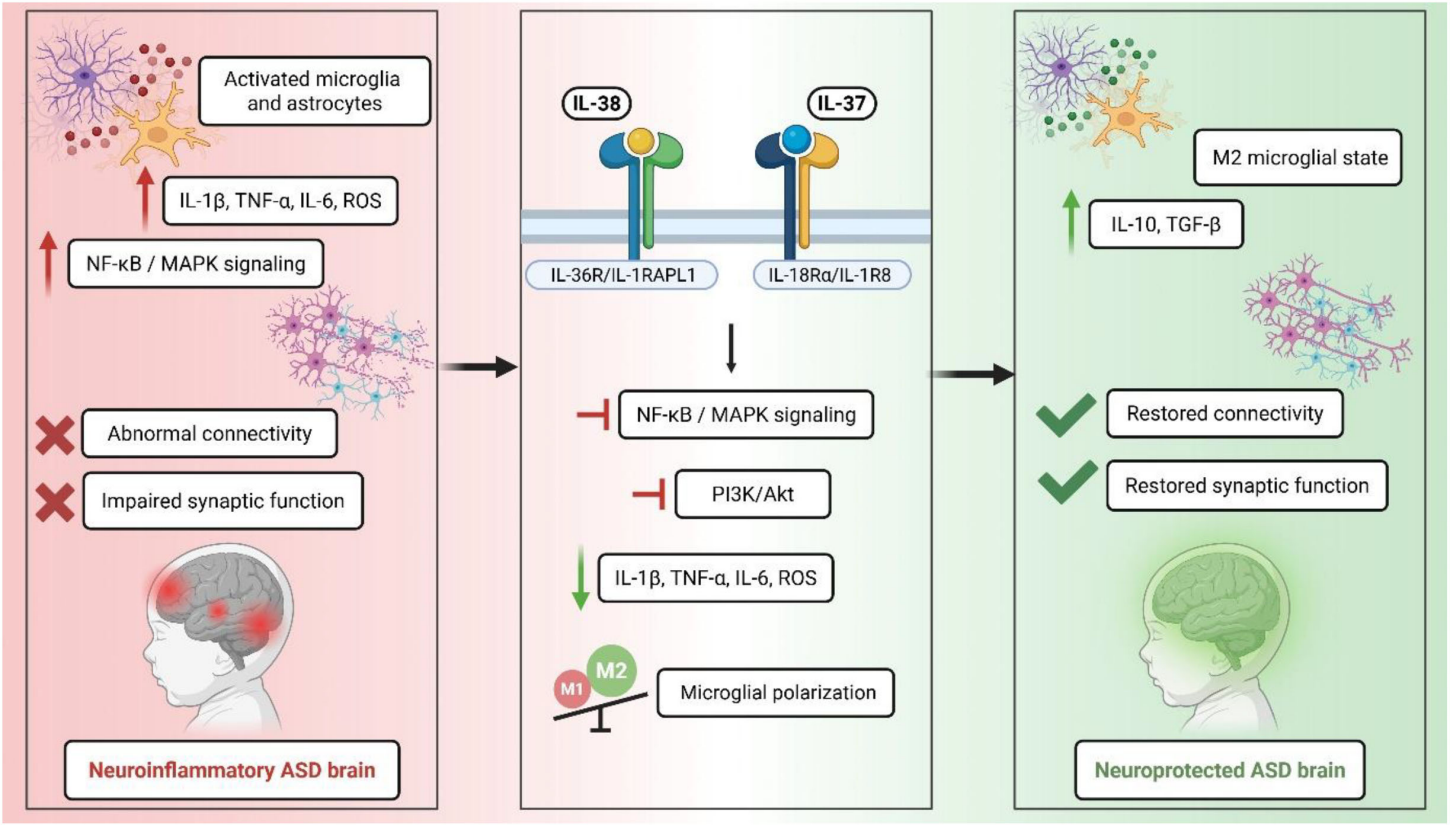
Conceptual framework illustrating how IL-37 and IL-38 modulation attenuates neuroinflammation and promotes neuroprotection in autism spectrum disorder (ASD). The left panel depicts the neuroinflammatory ASD brain, with activated microglia and astrocytes releasing IL-1β, TNF-α, IL-6, and ROS via NF-κB/MAPK signaling, leading to disrupted connectivity and synaptic dysfunction. The middle panel shows IL-37 and IL-38 engagement with IL-18Rα/IL-1R8 and IL-36R/IL-1RAPL1 receptors, suppressing NF-κB, MAPK, and PI3K/Akt pathways and promoting M1-to-M2 microglial polarization. The right panel represents the neuroprotected ASD brain, marked by anti-inflammatory M2 microglia, elevated IL-10 and TGF-β, reduced neuroinflammation, and restored neuronal connectivity and synaptic function.

Despite these obstacles, the potential of IL-37 and IL-38 cytokine research in autism is significant. The identification of a single biological target with evident therapeutic promise marks a substantial advancement in the development of precision medicine strategies for autism treatment (27, 67). As research in this domain progresses, the IL-37/38 axis may become a fundamental element of anti-inflammatory approaches for tackling the neuroinflammatory aspects of autism spectrum disease. This conceptual framework is summarized in **Figure 4**, which illustrates how IL-37 and IL-38 modulation can attenuate neuroinflammation, promote microglial homeostasis, and restore neuronal connectivity in ASD. The extensive implications of cytokine research exceed autism, encompassing additional neurodevelopmental and neuroinflammatory disorders. The principles and methodologies derived from IL-37 and IL-38 cytokine research may guide therapeutic methods for several illnesses characterized by neuroinflammation and immunological dysfunction (12, 134, 141).

In conclusion, this review exemplifies the potential of translational neuroscience to bridge the gap between fundamental scientific discoveries and clinical applications, which has the potential to enhance the quality of life for people who have autism and their families.
